# Cellular Immunotherapy for Prostate Cancer: Lessons Learned From 15 Years of Sipuleucel‐T

**DOI:** 10.1155/proc/9766669

**Published:** 2025-12-31

**Authors:** Neal D. Shore, Emmanuel S. Antonarakis, Jason Hafron, Kelvin A. Moses, Christopher Pieczonka, Benjamin Lowentritt, Nadeem Sheikh, Daniel J. George, Tanya Barauskas Dorff

**Affiliations:** ^1^ Genitourinary Oncology Center of Excellence, START Carolinas/Carolina Urologic Research Center, Myrtle Beach, South Carolina, USA; ^2^ University of Minnesota Masonic Cancer Center, University of Minnesota, Minneapolis, Minnesota, USA, umn.edu; ^3^ Clinical Research, Michigan Institute of Urology, West Bloomfield, Michigan, USA; ^4^ Department of Urology, Vanderbilt University Medical Center, Nashville, Tennessee, USA, vanderbilt.edu; ^5^ Clinical Research, Associated Medical Professionals of NY, Syracuse, New York, USA; ^6^ Minimally Invasive Surgery and Robotics, Chesapeake Urology, Towson, Maryland, USA; ^7^ Research and Manufacturing Science, Dendreon Pharmaceuticals, Seattle, Washington, USA; ^8^ Medical Oncology, Duke University Cancer Center, Durham, North Carolina, USA; ^9^ Department of Medical Oncology and Therapeutics Research, City of Hope National Cancer Center, Duarte, California, USA

**Keywords:** immunotherapy, prostate cancer, sipuleucel-T

## Abstract

The first cellular cancer immunotherapy, sipuleucel‐T, was approved for metastatic castration‐resistant prostate cancer (mCRPC) patients 15 years ago. Since then, the therapeutic landscape of advanced prostate cancer has significantly evolved. Sipuleucel‐T is a personalized, autologous immunotherapy that activates the patient’s immune system to target prostatic acid phosphatase (PAP)–expressing tumor cells and has demonstrated survival benefit in patients with nonopioid requiring mCRPC. Subsequent clinical trials and abundant real‐world data have provided further evidence of this novel immunotherapy’s clinical benefit for patients with mCRPC, as well as demonstrating the numerous immune and biologic responses that sipuleucel‐T induces. These data have also identified patient‐specific factors associated with longer survival, including race, baseline disease burden, and treatment‐induced immune responses. Despite the addition of multiple life‐prolonging therapeutic modalities now available to treat patients with mCRPC, the mechanism of action of sipuleucel‐T remains unique for patients with advanced prostate cancer. Therefore, maximizing the appropriate clinical utilization of sipuleucel‐T in patients with mCRPC within current treatment paradigms is essential.

## 1. Introduction

Before 2010, advanced prostate cancer management typically involved chronic androgen deprivation therapy followed by treatment with agents such as mitoxantrone or docetaxel chemotherapy in a subset of patients with symptomatic metastatic castration‐resistant prostate cancer (mCRPC) [1]. Thanks to intensified clinical research and investment, patient care for advanced prostate cancer has evolved significantly based on a greater understanding of cancer biology and novel tumor profiling [1, 2]. Multiple new agents targeting prostate cancer through different mechanisms of action have been approved, extending overall survival (OS) rather than merely delaying disease progression [1, 2]. A pivotal moment for this shift was the 2010 approval of sipuleucel‐T for patients with asymptomatic or minimally symptomatic mCRPC [3].

Sipuleucel‐T is a personalized, autologous treatment that was the first immunotherapy approved to treat solid tumors in the United States (USA) and Europe [3–5]. Sipuleucel‐T was designed to activate the patient’s immune system through ex vivo exposure to the target antigen prostatic acid phosphatase (PAP), resulting in cells of the immune system being able to detect and kill prostate cancer cells by recognition of PAP on prostate cancer cells [3, 6, 7]. In the registrational Phase III Immunotherapy for Prostate Adenocarcinoma Treatment (IMPACT) trial, sipuleucel‐T demonstrated a statistically significant improvement in median OS over placebo of 4.1 months (25.8 vs 21.7 months; hazard ratio [HR] = 0.78, *p* = 0.03) [8].

With its unique mechanism of action, sipuleucel‐T remains the only agent in its class and the only cellular immunotherapy proven to prolong OS in asymptomatic or minimally symptomatic mCRPC [3, 6]. However, estimates indicate that only 10% of eligible patients receive sipuleucel‐T treatment, suggesting that it is vastly underutilized in today’s therapeutic landscape [9, 10]. As we mark 15 years since the U.S. approval of sipuleucel‐T [3], we review the clinical and real‐world evidence beyond the initial data to better understand its clinical benefits in the context of contemporary prostate cancer management. Additionally, we highlight patient populations that may particularly benefit from sipuleucel‐T and discuss its optimal role within the modern landscape of advanced prostate cancer treatments.

## 2. Immunotherapy to Treat Prostate Cancer

The success of an anticancer immunotherapy relies on its ability to activate the patient’s immune system to allow for improved tumor control [11]. A challenge with prostate cancer is that its tumor microenvironment seems to exhibit limited T‐cell infiltration relative to other solid tumors [6, 12]. Except in the rare circumstance of microsatellite instability, immune checkpoint inhibitors have demonstrated little clinical benefit when used as monotherapy in mCRPC, possibly because they rely either on manipulating the activity of subsets of T cells already present in the tumor microenvironment [6, 12, 13] or on checkpoint inhibitor expression on tumor cells (Table 1) [22]. In contrast, sipuleucel‐T leverages the innate immune system to recruit T cells to the tumor microenvironment by ex vivo activation of antigen‐presenting cells (APCs) with PAP [6, 11, 13]. By activating an adaptive immune response independent of the tumor microenvironment, sipuleucel‐T appears capable of eliciting immunologic memory against the commonly expressed PAP antigen [23].

**Table 1:** Previous registrational Phase III trials for immunotherapies in prostate cancer.

Treatment	MoA	Phase	Design	Outcome
GVAX [14]	Allogeneic GM‐CSF‐secreting tumor cell–based immunotherapy	VITAL‐1 (Phase III) VITAL‐2 (Phase III)	GVAX vs SOC (docetaxel plus prednisone) in asymptomatic mPC	Terminated (2008)
PROSTVAC‐VF [15] (a recombinant vaccinia virus [rilimogene galvacirepvec] and a recombinant fowlpox virus [rilimogene glafolivec])	Active immunotherapy vaccine that contains PSA as the tumor‐associated antigen used to generate a T‐cell response against prostate cancer	Phase III	PROSTVAC plus GM‐CSF vs PROSTVAC plus placebo GM‐CSF vs vaccine placebo plus placebo GM‐CSF	Clinical development stopped for monotherapy
Ipilimumab [16]	Anti‐CTLA4	Phase III	Ipilimumab monotherapy vs placebo (pre‐ and postchemotherapy)	No improvement in OS in mCRPC patients
Pembrolizumab plus olaparib [17]	Anti‐PD1; PARP inhibitor	Phase III open label	Pembrolizumab plus olaparib vs abiraterone or enzalutamide	No improvement in rPFS or OS in mCRPC; terminated
Pembrolizumab [18]	Anti‐PD1	Phase III	Pembrolizumab plus docetaxel vs placebo vs docetaxel	No improvement in efficacy outcomes in mCRPC
Pembrolizumab [19]	Anti‐PDI	Phase III	Pembrolizumab plus enzalutamide vs placebo plus enzalutamide	No improvement in efficacy outcomes in mCRPC
Pembrolizumab [20]	Anti‐PDI	Phase III	Pembrolizumab plus enzalutamide plus ADT vs placebo plus enzalutamide plus ADT	No improvement in rPFS or OS in mHSPC; terminated
DCVAC/PCa [21]	Active cellular immunotherapy based on autologous dendritic cells to activate antitumor response	Phase III	DCVAC/PCa plus docetaxel plus prednisone vs placebo plus docetaxel plus prednisone	No improvement in OS in mCRPC

Abbreviations: ADT, androgen deprivation therapy; CTLA‐4, cytotoxic T‐lymphocyte‐associated protein 4; GM‐CSF, granulocyte–macrophage colony‐stimulating factor; mCRPC, metastatic castration‐resistant prostate cancer; mHSPC, metastatic hormone‐sensitive prostate cancer; MoA, mechanism of action; mPC, metastatic prostate cancer; OS, overall survival; PARP, poly(ADP‐ribose) polymerase; PD1, programmed cell death protein 1; PSA, prostate‐specific antigen; rPFS, radiographic progression‐free survival; SOC, standard of care.

**Figure 1:**
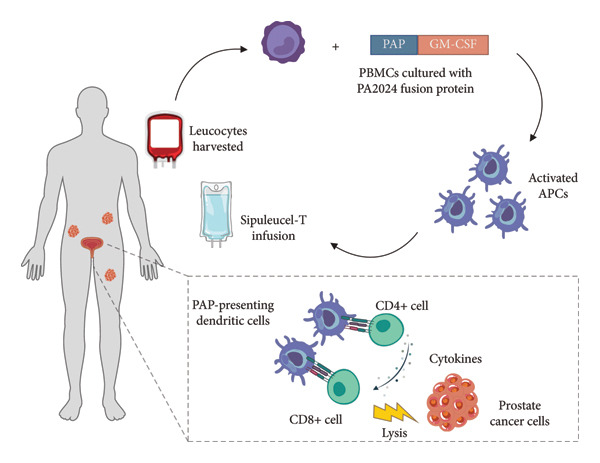
Mechanism of action of sipuleucel‐T [6, 24]. APC, antigen‐presenting cell; CD, cluster of differentiation; GM‐CSF, granulocyte–macrophage colony‐stimulating factor; PAP, prostatic acid phosphatase; PBMC, peripheral blood mononuclear cells.

Sipuleucel‐T treatment consists of three cycles, each comprising three steps: apheresis, isolation of the patient’s peripheral blood mononuclear cells (PBMCs) and subsequent activation of the APCs by exposure to PA2024, and reinfusion of the activated cells (Figure 1) [3, 7]. PA2024 is a recombinant fusion protein that combines the prostate‐specific PAP antigen with granulocyte–macrophage colony‐stimulating factor (GM‐CSF), a cytokine that activates APCs [3, 25]. On reinfusion, the patient’s immune system generates PA2024‐specific and PAP‐specific cellular and humoral responses [3, 6].

## 3. Mechanisms of Action of Sipuleucel‐T

The recognition and destruction of a foreign antigen by immune cells is the initial result of antigen uptake and subsequent activation of APCs. These APCs, in turn, present antigens in the form of peptides via Class I and Class II major histocompatibility complexes to T cells, which are the effector cell component of the innate immune system [26]. With sipuleucel‐T therapy, ex vivo exposure to PA2024 trains the patient’s immune system to respond to prostate cancer cells in a similar manner by educating T cells to recognize PAP [6]. Analysis of patients who received sipuleucel‐T in the Phase III IMPACT trial demonstrated serial activation of APCs during the manufacture of sipuleucel‐T [27]. This analysis found evidence of a prime‐boost effect with each subsequent sipuleucel‐T infusion, resulting in cumulative APC activation [27]. Sipuleucel‐T administration induced not only peripheral cellular response, but also humoral immune responses, which were identified in 79% of sipuleucel‐T‐treated patients in IMPACT compared with 13% of patients in the control group [27]. In both the IMPACT trial and the subsequent PROVENGE Registry for the Observation, Collection, and Evaluation of Experience Data (PROCEED) study, key indicators of immune activation and product potency (cumulative APC activation, APC count, and total nucleated cell count) were significantly positively correlated with OS [27–29].

In vitro activity of activated T cells was recently captured using microscopy from sipuleucel‐T‐treated patient PBMCs, providing direct visualization of sipuleucel‐T‐induced T cells killing PAP‐expressing cells. [30]. Induction of cytotoxic T lymphocytes that kill cells expressing the target antigens has also been demonstrated, with these cytotoxic T lymphocyte responses at 26 weeks after sipuleucel‐T treatment also associated with improved OS [31]. Elevated immunoglobulin G (IgG) levels against multiple tumor‐specific, nontargeted (secondary) antigens, including prostate‐specific antigen (PSA), LGALS3, and ERAS, were identified in patients with mCRPC by 2 weeks after sipuleucel‐T treatment [32]. These data suggest an expansion of the immune response, referred to as humoral antigen spread, that appears to result from the destruction of targeted tumor cells [32]. These IgG responses were associated with OS, suggesting that a greater extent of antigen spread may improve OS [32]. Development of long‐term antigen‐specific immunological memory has also been suggested based on higher APC activation and altered B‐cell receptor repertoire in men retreated with sipuleucel‐T years after their initial treatment compared with sipuleucel‐T‐naïve patients [23, 33].

Sipuleucel‐T also induces local immune effects that act at the prostate tissue–tumor interface. As mentioned, prostate cancer has typically been characterized as “cold,” with little immune activity within the tumor [6]. Treatment with sipuleucel‐T has been shown to induce the movement of activated T cells (i.e., trafficking) to the stromal–epithelial cell interface within the tumor microenvironment [34]. This trafficking was observed after neoadjuvant administration of sipuleucel‐T before radical prostatectomy in the Phase II Neo‐ACT study [34] and was associated with a dynamic change in T‐cell receptor diversity within the tumor microenvironment [35].

A comprehensive review of these mechanisms of action was recently published, providing detailed evidence for sipuleucel‐T inducing both systemic and localized immune responses [6]. Collectively, the data suggest that sipuleucel‐T elicits a multifactorial and persistent antitumor immune response that correlates with survival benefit [6, 13].

## 4. Clinical Evidence for Sipuleucel‐T in Patients With Advanced Prostate Cancer

When the sipuleucel‐T Phase III trials were initiated, the goals for the standard of care for mCRPC typically focused on delaying disease progression, with a median OS of 12–22 months reported in Phase III clinical trials of other therapies [36–41]. Before 2010, the chemotherapeutic agent docetaxel was the only approved therapy shown to prolong survival in mCRPC, conferring a median survival benefit of 2–3 months over chemotherapy with mitoxantrone plus prednisone, which was primarily used for pain palliation [36, 38, 39].

Sipuleucel‐T demonstrated an OS benefit across all three of its Phase III, randomized, placebo‐controlled trials in men with mCRPC [8, 42, 43]. The first two Phase III studies were designed with a primary endpoint of delayed time to disease progression; notably, neither met their primary endpoints nor showed an OS benefit [42, 43]. Based on these consistent results, a prospective, double‐blind, placebo‐controlled Phase III trial (IMPACT) was designed to appropriately assess OS as the primary endpoint. IMPACT randomized 512 men with asymptomatic or minimally symptomatic mCRPC and demonstrated a 22% reduction in risk of death (HR = 0.78, 95% confidence interval [CI]: 0.61–0.98, *p* = 0.03 vs placebo), representing a median survival benefit of 4.1 months (OS of 28.5 months vs 21.7 months with placebo) assessed after a median follow‐up time of 34.1 months [8]. As observed previously, sipuleucel‐T was not associated with prolongation of progression‐free survival or time to clinical progression, which may reflect the kinetics of immune activation by sipuleucel‐T leading to delayed antitumor response [8]. Based on these results, sipuleucel‐T was approved for use by the U.S. Food and Drug Administration (FDA) in men with asymptomatic or minimally symptomatic mCRPC on April 29, 2010.

The novel and innovative nature of sipuleucel‐T raised criticism from some physicians, as there was concern that the placebo arm may hypothetically compromise immune function [44, 45]. Subsequent analyses did not support this concern, which showed that white blood cell counts remained stable throughout treatment and that both treatment arms exhibited similar rates of infections [44, 46, 47].

One feature of IMPACT’s study design that may have impacted the observed OS benefit of 4.1 months was the crossover design, which permitted patients in the placebo arm to receive salvage therapy upon objective disease progression [8]. Almost 64% of placebo‐treated patients received therapy (49% as the first salvage intervention) with APC8015F, an autologous cell product similar to sipuleucel‐T but produced using cells cryopreserved at the time the placebo treatment was prepared [8]. An exploratory analysis that examined the impact of this crossover suggested a positive OS benefit with APC8015F salvage therapy ranging from 4.1 to 7.8 months, depending on the assumed effectiveness of APC8015F relative to sipuleucel‐T [48].

The positive outcome of IMPACT on OS led to U.S. FDA approval in 2010 for sipuleucel‐T to treat men with asymptomatic to minimally symptomatic mCRPC, making it the first immunotherapy agent to treat a solid cancer [3, 4]. From this point, evaluations of subsequent cancer therapeutics included survival endpoints. Sipuleucel‐T was also approved by the European Medicines Agency for use in Europe in 2013 but was later withdrawn from the European market in 2015 at the manufacturer’s request [5, 49].

The postapproval prospective PROCEED registry study provided key effectiveness data on patients who were treated with commercial sipuleucel‐T for the approved indication and then followed from 2011 to 2017 [28]. Across 1976 men in PROCEED treated with sipuleucel‐T and followed for a median of 46.6 months, OS was 30.7 months (95% CI: 28.6–32.2), with 1‐year and 2‐year treatment‐free intervals achieved by 32.5% and 17.4% of patients, respectively [28]. Delayed PSA responses have been observed in an exploratory analysis of the PROCEED registry and retrospective cohort review of Dana‐Farber Cancer Institute patients who did not initiate a new therapy for at least 6 months after completion of sipuleucel‐T treatment [50]. This study observed that 14%–20% of patients achieved a 50% decline in PSA from baseline at a median of 5.5–6.3 months [50]. Similarly, another retrospective study identified a subset of patients (4%–13%) with PSA stabilization for at least 2 months after completing sipuleucel‐T treatment and a median of 17.8 months to subsequent therapy [51]. These real‐world data support the efficacy of sipuleucel‐T despite the lack of significant progression‐free survival benefit observed in the Phase III clinical trials.

Over 40,000 patients have now received sipuleucel‐T treatment. Both clinical and real‐world data demonstrate a safety and tolerability profile consistent with what was observed during the Phase III studies [8, 28, 47, 52]. Adverse events are predominantly mild to moderate, with transient infusion‐related reactions, chills, pyrexia, fatigue, headache, nausea, myalgia, and influenza‐like illness reported at higher rates in sipuleucel‐T‐treated patients than in controls [8, 28, 47, 52]. Patients requiring placement of an apheresis catheter face additional risks. In a pooled analysis of the Phase III trials, rates of cerebrovascular events (CVEs) were slightly higher in patients treated with sipuleucel‐T (3.5%) than with placebo (2.6%) [47]. However, subsequent data from the PROCEED registry show a CVE rate of 2.8%, which is the same as that found in the Surveillance, Epidemiology, and End Results (SEER)–Medicare database across 11,972 men with mCRPC [28]. Unlike other immunotherapies, sipuleucel‐T is not associated with significant autoimmune toxicities, cytokine release syndrome, immune effector cell–associated neurotoxicity syndrome, or liver or renal dysfunction [8, 28, 47, 52].

## 5. Evolving Diagnostic and Treatment Paradigms for Prostate Cancer

Our understanding of prostate cancer treatment response has evolved considerably over the past 15 years. Terminology has changed from hormone‐resistant to castration‐resistant, recognizing that the androgen receptor remains a disease driver in advanced prostate cancer [53]. The approval of therapeutic agents targeting this pathway, such as abiraterone and enzalutamide, after the approval of sipuleucel‐T raises questions about treatment sequencing [54]. Additionally, these agents—which were initially developed and approved for mCRPC—are now used in metastatic hormone‐sensitive prostate cancer (mHSPC) [55]. In addition, characterization of molecular features of mCRPC has led to the development of targeted therapies, such as poly(ADP‐ribose) polymerase inhibitors (PARPis) and 177‐lutetium–PSMA‐617, which are also showing promise in the mHSPC setting [56–58]. This is creating a different mCRPC phenotype in a subset of patients that affects further treatment options [55]. Furthermore, defining and managing metastatic prostate cancer is transforming in the context of enhanced imaging technologies and a greater understanding of the complex relationship between serum PSA levels and prostate cancer [59]. This shift is reflected in the changing natural history of prostate cancer incidence, prevalence, and mortality observed in the United States [60].

As rising PSA levels have become less important for defining disease progression, PSA levels at treatment initiation are increasingly recognized as a marker of disease burden and a predictor of response to therapy (Box 1). Exploratory analyses of the IMPACT study identified PSA levels as the strongest baseline prognostic factor for survival benefit with sipuleucel‐T (*p* < 0.0001) [29]. Lower baseline PSA levels improved OS outcomes, with an estimated median OS of 13.0 months (HR = 0.51, 95% CI: 0.31–0.85) for men in the lowest PSA quartile (≤ 22 ng/mL) and 2.8 months (HR = 0.84, 95% CI: 0.55–1.29) for men in the highest PSA quartile (> 134 ng/mL) [29]. This association was independently validated in a post hoc analysis of PROCEED registry data: Median OS was 47.7 months for men in the lowest baseline PSA quartile (≤ 5.27 ng/mL) compared with 33.2 months, 27.2 months, and 18.4 months for men in the second (> 5.27 to ≤ 15.08 ng/mL), third (> 15.08 to ≤ 46 ng/mL), and fourth (> 46 ng/mL) PSA quartiles, respectively [28].

**Box 1: Predictors of response to sipuleucel‐T treatment.**
 Lower levels of PSA at treatment initiation [28, 29]
 Race (increased survival in Black men) [61–65]
 Preexisting immune status (postulated) [28, 51, 66, 67]

Lower PSA levels generally indicate a reduced disease burden, which correlates with less systemic and tumor microenvironment immunosuppression [29, 68]. As sipuleucel‐T treatment depends on activating an immune response, the greater OS benefit associated with lower PSA levels suggests that treating patients earlier in their disease course may provoke a more effective and potent immune response than treating those with a higher disease burden [29]. Enhanced immune activity in earlier disease stages is supported by data from patients enrolled in clinical trials of sipuleucel‐T, which shows a greater magnitude of APC activation in the neoadjuvant setting than in the metastatic treatment setting [69].

## 6. Predictors of Sipuleucel‐T Response

Race emerged as another factor that predicted response to sipuleucel‐T after analyses of data pooled from the Phase III sipuleucel‐T studies identified a significant survival benefit in Black men (Box 1) [61–63]. Longer median OS was observed in Black men (45.3 months, *n* = 33) than in the overall population (25.4 months, *n* = 488) [61]. A greater increase in median OS with sipuleucel‐T vs control was observed in the Black subpopulation (30.7‐month difference) than in the overall population (3.9‐month difference) [61]. The OS benefit remained when those 33 Black men were matched 2:1 for baseline characteristics with White men, with a 20.6‐month difference (45.3 months vs 24.7 months; HR = 0.49, 95% CI: 0.26–0.91, *p* = 0.02) [63]. The number needed to benefit (preventing one additional death vs placebo) was lower for Black men than the overall population at both 24 months (5 vs 10) and 36 months (3 vs 8) [62]. The declining number over time suggests the durability of clinical benefit in both groups assessed [62].

The survival benefit of sipuleucel‐T in Black men was also noted in independent postapproval real‐world studies [64, 65]. An exploratory analysis of the PROCEED registry, which included higher representation of Black men (11.7%) than the Phase III trials (6.7%), identified race as a significant independent predictor of OS after sipuleucel‐T treatment (HR = 0.81, 95% CI: 0.68–0.97, *p* = 0.03) [63, 65]. In this analysis, median OS was longer in Black men (35.2 months vs 29.9 months in White men) treated with sipuleucel‐T [63, 65]. As baseline PSA levels (a significant predictor of OS with sipuleucel‐T) were significantly higher in the Black PROCEED patients, an analysis of a baseline PSA‐matched cohort (2:1 White:Black patients) was performed [65]. This analysis also demonstrated longer median OS in Black men (35.3 months vs 25.8 months in PSA‐matched White men; HR = 0.70, 95% CI: 0.57–0.86, *p* < 0.0001) [65]. A retrospective analysis of Black men in a Medicare Fee‐for‐Service population identified that 76% of patients treated with sipuleucel‐T (*n* = 140) were alive at 24 months compared with 49% of patients treated with oral agents (*n* = 1266) [64]. In this population, sipuleucel‐T use was associated with a 63% reduction in risk of death (HR = 0.37, *p* < 0.0001 vs no sipuleucel‐T) [64]. In a single‐institution retrospective analysis of men with mCRPC treated with sipuleucel‐T, Black men represented 30%–50% of the subset of patients who exhibited post‐treatment PSA stabilization despite comprising only 20%–24% of the total study population [51].

These are important findings given that Black men are disproportionately affected by prostate cancer and are typically underrepresented in clinical trial populations [70]. Incidence of prostate cancer has been reported as 1.8 times higher in Black men than White men, with a prostate cancer‐specific mortality rate 2.2 times higher [71, 72]. In clinical trials with sipuleucel‐T in the mCRPC setting, Black men exhibited similar or better outcomes than White men, for reasons that are not completely understood [70]. Underlying differences in immune phenotypes and both innate and adaptive immune activity between Black and non‐Black populations may in part explain the racial differences observed in response to sipuleucel‐T [6, 73]. These reported immune differences include proportions of circulating immune cells, expression of genes regulating inflammatory cytokines, and B‐cell and T‐cell signaling [6, 73]. Racial differences in the tumor microenvironment in prostate cancer are also implicated, with evidence of higher expression of inflammatory cytokines and lymphocytic infiltrates in Black men than in non‐Black men [73]. This may suggest that the tumor microenvironment is more immunogenic in Black men than in non‐Black men, which may enhance their response to immunotherapies [73].

Recent studies have compared immune parameters between Black and White men with mCRPC treated with sipuleucel‐T to help identify differences in treatment responses that may explain survival outcomes. Assessment of circulating immune markers in patients enrolled in the PRIME immune monitoring registry, a subset of PROCEED, showed that Black men (*n* = 18) had higher median concentrations of Th2‐type and inflammatory cytokines than PSA‐matched White men (*n* = 36) at baseline and at 52 weeks after sipuleucel‐T treatment (*p* < 0.05) but not at treatment completion (Week 6) or Week 26 [74]. This may suggest that the Black men experienced higher systemic inflammation that was transiently attenuated by sipuleucel‐T [74]. A single‐arm study also identified serum levels of inflammatory cytokines and expression of costimulatory molecules on T cells before and after treatment with sipuleucel‐T that were higher in Black men (*n* = 29) than in non‐Black men (*n* = 28) [75]. Neither of these studies observed differences between groups in PA2024 or PAP antigen‐specific cellular and humoral immune responses and did not identify any associations between immune responses and OS [74, 75]. The authors of these studies note that these latter findings may reflect levels of circulating immune biomarkers that do not accurately represent the tumor microenvironment [74, 75]. Another study found similar peripheral immune responses, with higher T‐cell and B‐cell responses in Black men (*n* = 10) than in White men (*n* = 20) but similar humoral responses to PA2024 and PAP antigens after sipuleucel‐T treatment [66].

Currently, there are no biomarkers to measure or predict sipuleucel‐T response, although these early data suggest variations based on immune status (i.e., a Th‐2 immune phenotype) may help stratify patient responses (Box 1). Evidence from the PROCEED registry suggests that a subset of patients may experience an extended response to sipuleucel‐T treatment: Of the 77.1% of patients who received one or more subsequent lines of life‐prolonging therapy, 32.5% and 17.4% experienced 1‐year and 2‐year treatment‐free intervals, respectively [28]. Further characterization of patients with similar responses, as well as those who experience stabilization or even decline in PSA after sipuleucel‐T treatment [51], may identify specific immune biomarkers that could help predict which patients are most likely to respond well to sipuleucel‐T with sustained clinical benefits.

Another factor to consider when selecting patients for sipuleucel‐T treatment is the potential impact of prior lines of therapies on preexisting immune status and subsequent responsiveness to sipuleucel‐T. This effect was observed in an open‐label Phase II trial of a combination of sipuleucel‐T and ipilimumab, where a history of radiation therapy was associated with lower frequencies of circulating CTLA‐4‐positive T cells and good clinical outcomes [76].

## 7. Considerations for Use of Sipuleucel‐T in the Current mCRPC Treatment Landscape

For over a decade, sipuleucel‐T has been recommended as a first‐line treatment for patients with asymptomatic and minimally symptomatic mCRPC in multiple guidelines, including the National Comprehensive Cancer Network (Category 1 recommendation), the American Urological Association, and the American Society of Clinical Oncology [77–81]. The past decade has also seen the approval of nine additional life‐prolonging agents for mCRPC in the United States—all with distinct modes of action (Figure 2) [54, 82].

Figure 2(a) Timeline of approvals and (b) mechanisms of action for FDA‐approved therapeutic agents for treating mCRPC [54, 82]. APC, antigen‐presenting cell; BRCA, breast cancer gene; CD, cluster of differentiation; dMMR, mismatch repair deficient; DNA, deoxyribonucleic acid; HRR, homologous recombination repair; MHC, major histocompatibility complex; MSI‐H, microsatellite instability—high; PAP, prostatic acid phosphatase; PARP, poly (ADP‐ribose) polymerase; PD‐L1, programmed death ligand 1; PSMA, prostate‐specific membrane antigen.

**Figure:**
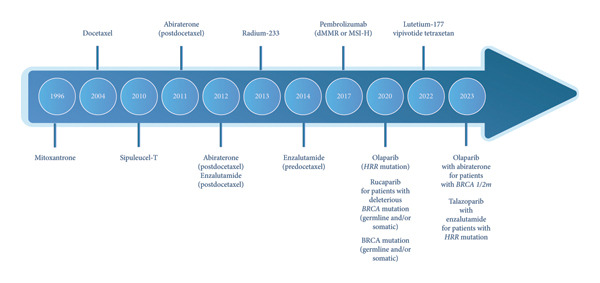
(a)

**Figure:**
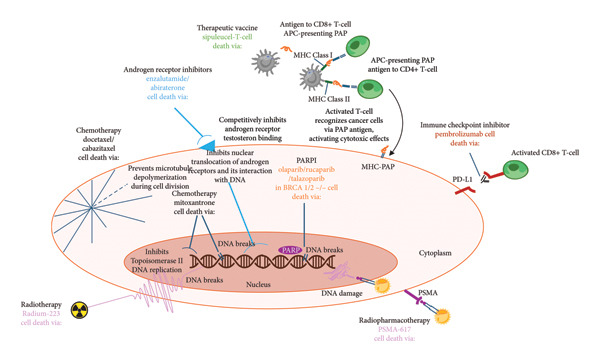
(b)

Despite the increased availability of treatment options, real‐world studies consistently indicate low utilization of life‐prolonging agents in men with mCRPC. A retrospective analysis of 14,780 men in a Medicare Fee‐for‐Service claims database showed that only 78% of patients received one or more lines of life‐prolonging therapies after mCRPC diagnosis, and fewer than 50% received at least two classes of treatments [83]. Similarly, a retrospective electronic health record analysis of 2559 patients with mCRPC showed that 77% received at least one line of life‐prolonging therapy, with only 49% of those receiving a second line and 43% of those receiving a third line [9]. Across both studies, monotherapy with the androgen receptor pathway inhibitors (ARPIs) abiraterone and enzalutamide was most commonly used, accounting for over 50% of all first‐ and second‐line therapies [9, 83]. Importantly, ARPIs are frequently used sequentially, with 25% of patients who received first‐line abiraterone receiving second‐line enzalutamide and 17% who received first‐line enzalutamide receiving second‐line abiraterone [9]. Sipuleucel‐T was considered underutilized across all lines of therapy, with use highest as a first‐line therapy in only 7% of patients [9]. This is consistent with the 11% first‐line use of sipuleucel‐T seen in a Medicare claims database of 6044 chemotherapy‐naïve men treated for mCRPC with sipuleucel‐T or ARPIs [10].

The proliferation of mCRPC treatment options underscores the growing role of precision medicine in advanced prostate cancer, where therapies can be targeted to specific molecular or biologic characteristics of individual patients [84, 85]. However, increasing use of many of these agents in earlier disease settings raises challenges for identifying optimal sequencing and combinations of therapies for patients with mCRPC [54, 81]. Most patients are treated with sequential ARPI monotherapy despite evidence suggesting limited clinical benefit due to relatively high cross‐resistance [9, 86, 87]. Guidelines now recommend using agents with differing mechanisms of action, and efforts are ongoing to refine existing treatment practices through strategic therapeutic layering of approved agents [54, 78, 81]. Studies examining the effects of sequential and concurrent use of sipuleucel‐T with a number of these agents are helping guide the position and optimal timing of sipuleucel‐T within the mCRPC treatment landscape (Table 2).

**Table 2:** Clinical studies for optimizing sipuleucel‐T in practice.

Regimen	Study name	Outcomes
Treatment during active surveillance	ProVENT [88]	Preliminary analysis suggests higher sipuleucel‐T potency in active surveillance [88]

In combination with radiopharmacotherapy/radiation therapy	Study of sipuleucel‐T with or without radium‐223 in men with asymptomatic or minimally symptomatic bone‐mCRPC [89]	Superior clinical activity with combination despite greater PA2024‐specific T‐cell responses with sipuleucel‐T monotherapy [89]
	Sipuleucel‐T and stereotactic ablative body radiation (SABR) for mCRPC [90]	Humoral and cellular immune responses induced but no clear synergistic benefit with concurrent treatment [90]
	Radiation therapy in treating patients with metastatic hormone‐resistant prostate cancer receiving sipuleucel‐T [91]	No effect of radiation therapy on sipuleucel‐T product but no enhancement of cellular or humoral immune responses [91]

In combination with another immunotherapy	Sipuleucel‐T with immediate vs delayed CTLA‐4 blockade for prostate cancer [76]	Combination was well‐tolerated, and timing of ipilimumab did not affect immune response to sipuleucel‐T [76]
	Phase Ib study of patients with metastatic castrate‐resistant prostate cancer treated with different sequencing regimens of atezolizumab and sipuleucel‐T	Safety profile comparable to monotherapies, regardless of order given; immune studies suggest possible benefit from combination but objective response rate only 4.3% [92]

In combination with DNA vaccine	Sipuleucel‐T with or without pTVG‐HP DNA booster vaccine in prostate cancer [93]	Prime‐boost vaccination can augment and diversify T‐cell and humoral immunity elicited by antitumor vaccination [93]

In combination with androgen receptor inhibitor therapy	Combination treatment with sipuleucel‐T and abiraterone acetate or enzalutamide for metastatic castration‐resistant prostate cancer: STAMP and STRIDE trials [94]	Combinations can be used safely without compromising immune activity of sipuleucel‐T or subsequent OS benefit [94]

Extended treatment	An extended course of sipuleucel‐T immunotherapy for treating patients with metastatic castration‐resistant prostate cancer, EXCITE trial [95]	Study in recruitment phase

*Note:* CLTA‐4, cytotoxic T‐lymphocyte‐associated protein 4; DNA, deoxyribonucleic acid; pTVG‐HP, plasmid DNA encoding human prostatic acid phosphatase.

Abbreviations: mCRPC, metastatic castration‐resistant prostate cancer; SABR, stereotactic ablative body radiation.

As sipuleucel‐T approval was based on OS benefit and not progression‐free survival, there is an opportunity to utilize sipuleucel‐T as a lead‐in to subsequent treatment—without waiting for disease progression. A complete treatment course of sipuleucel‐T can be completed in 4 weeks and is not associated with delayed or lingering adverse events [3], further supporting its efficient use before subsequent treatment. These factors also highlight the cost‐effectiveness of sipuleucel‐T for treating mCRPC in the current landscape. The number‐needed‐to‐treat benefit is lower with sipuleucel‐T than with abiraterone and enzalutamide, and sipuleucel‐T demonstrates lower attendant costs associated with adverse drug events than other treatments including docetaxel, radium‐233, abiraterone, and enzalutamide [96, 97]. Overall attendant costs are also lower with sipuleucel‐T than with abiraterone and enzalutamide, which is due to the short window needed to complete sipuleucel‐T treatment compared with the ongoing administration required for treatment effect with ARPIs [96, 98].

Given the high utilization of ARPIs as first‐line treatments, a greater understanding of the potential effects of abiraterone and enzalutamide on immune activity and survival outcomes with sipuleucel‐T treatment is important [9, 10, 83]. Real‐world evidence supporting the clinical benefit of sipuleucel‐T and ARPI combinations was observed in two retrospective Medicare claims database analyses (Table 3) [10, 98]. Among 6044 men treated for mCRPC, the use of any line sipuleucel‐T was associated with improved median OS (35.2 months) compared to ARPI use alone (20.7 months; adjusted HR = 0.59, 95% CI: 0.53–0.65) [10]. First‐line sipuleucel‐T also demonstrated longer OS than first‐line ARPI (34.9 vs 21.0 months; adjusted HR = 0.56, 95% CI: 0.49–0.63) [10]. Combination therapy with sipuleucel‐T plus an ARPI in either sequence (*n* = 733; median OS 30.4 months) was associated with a 28.3% lower risk of death (HR = 0.72, 95% CI: 0.65–0.79, *p* < 0.01) than ARPI monotherapy (*n* = 4642; median OS 14.3 months) [98]. To investigate the effects of layering sipuleucel‐T with an ARPI, two Phase II, randomized, open‐label studies assessed the effects of sequential vs concurrent administration of sipuleucel‐T with abiraterone (STAMP study; *n* = 69 men) or sipuleucel‐T with enzalutamide (STRIDE study; *n* = 52 men) [94]. Long‐term follow‐up data showed no differences in median OS between sequential (sipuleucel‐T then ARPI) and concurrent treatment (33.3 months in STAMP and 32.5 months in STRIDE) within each study or across both studies [94]. Neither ARPI appeared to interfere with sipuleucel‐T‐induced APC activation, and no additive toxicity was observed [94].

**Table 3:** Survival outcomes for combination therapy with sipuleucel‐T and androgen receptor pathway inhibitors in patients with mCRPC.

Regimen	Improvement in median OS with sipuleucel‐T (months)	Hazard ratio
Sipuleucel‐T (any line of therapy) vs ARPI monotherapy [10]	14.5	0.59 (95% CI: 0.53–0.65)
Sipuleucel‐T (first line) vs ARPI (first line) [10]	13.9	0.56 (95% CI: 0.49–0.63)
Sequential sipuleucel‐T + ARPI or ARPI + sipuleucel‐T vs ARPI monotherapy [98]	16.1	0.72 (95% CI: 0.65–0.79)

Abbreviations: ARPI, androgen receptor pathway inhibitor; CI, confidence interval; mCRPC, metastatic castration‐resistant prostate cancer; OS, overall survival.

Collectively, these data highlight the opportunity for use of sipuleucel‐T earlier in the treatment paradigm, especially for patients with prior ARPI use in the first‐line mCRPC or nonmetastatic CRPC setting. As more agents originally developed for mCRPC become approved in earlier disease settings (e.g., PARPis and 177‐lutetium‐PSMA‐617) [56, 58], sipuleucel‐T may be increasingly considered for first‐line mCRPC treatment. Importantly, its high tolerability and short treatment time do not preclude the use of other approved agents [3]. In addition, potential synergistic benefits of sipuleucel‐T with other mechanisms of action support layering its use with other treatments. As described above, sequential treatment with sipuleucel‐T and an ARPI in either sequence has demonstrated survival benefit over ARPI alone, and those benefits are similar to and even concurrent use of these agents [10, 94, 98]. Ongoing combination or layering studies of sipuleucel‐T with ipilimumab [10, 76, 98, 99], atezolizumab [100], radiation therapy [90, 91], and radium‐233 [89] are all avenues of current investigation (Table 2). There are currently no studies evaluating sipuleucel‐T as a component of triplet therapy for mCRPC.

## 8. Conclusion

For 15 years, sipuleucel‐T was the only cellular immunotherapy available and remains one of only two life‐prolonging cellular immunotherapies for treating solid tumors [8, 101]. With its unique mechanism of action, it remains the only agent in its class and the only immunotherapy with proven survival benefit in asymptomatic or minimally symptomatic mCRPC [3, 6]. Yet today, sipuleucel‐T is underutilized, with only an estimated 10% of eligible patients receiving sipuleucel‐T treatment [9, 10]. Given recent work that highlights the likelihood that prostate cancer is undertreated in the United States, considering how and when each therapeutic agent in the prostate cancer armamentarium can be used helps us provide patients with the best treatment options [83, 102]. Since the approval of sipuleucel‐T 15 years ago, we have made significant progress in characterizing the immune mechanisms driving the observed survival benefit [6, 13], as well as in identifying and characterizing patients who are most likely to benefit from sipuleucel‐T treatment [6, 8, 13, 28, 29, 65]. Notably, greater survival benefit is observed in patients with lower disease burden, and evidence suggests variations in underlying immune phenotypes may help identify biomarkers to predict sipuleucel‐T responders [74, 75]. Clinical trials and real‐world evidence support the clinical benefit of initiating sipuleucel‐T early in the mCRPC disease course, with improved OS seen when using a combination of sipuleucel‐T and ARPIs—agents that are preferentially used as sequential first‐line monotherapy [10, 94, 98]. Investigation of similar strategic therapeutic layering of sipuleucel‐T and other agents with diverse mechanisms of action may provide further avenues for improving outcomes for patients with mCRPC in the future.

## Conflicts of Interest

This work was sponsored by Dendreon. Dendreon was involved in the design, review, preparation, and approval of and decision to submit the manuscript for publication.

N.D.S. received consulting/research fees from Alessa, Amgen, Astellas, AstraZeneca, Bayer, BMS, Daiichi Sankyo, Dendreon Pharmaceuticals, Janssen, Lilly, Merck, Photocure, Pfizer, Sumitomo, Tolmar, and Tutelix.

E.S.A. received grants and personal fees from Janssen, Sanofi, Bayer, Bristol Myers Squibb, Curium, MacroGenics, Merck, Pfizer, AstraZeneca, and Clovis; received personal fees from Aadi Bioscience, Aikido Pharma, Astellas, Amgen, Blue Earth, Boundless Bio, Corcept Therapeutics, Dendreon Pharmaceuticals, Exact Sciences, Hookipa Pharma, Invitae, Eli Lilly, Foundation Medicine, Menarini Silicon Biosystems, Tango Therapeutics, Tempus, and Z‐alpha; received grants from Novartis, Celgene, and Orion; and has a patent for an AR‐V7 biomarker technology that has been licensed to Qiagen.

J.H. reports the following roles: consultant/advisor for Astellas, AstraZeneca, Dendreon Pharmaceuticals, Immunis.AI, Janssen, Lipella Pharmaceuticals, Myriad Genetics, Pfizer, Photocure, Sumitomo Pharma, Tolmar Pharmaceuticals, and UroGen Pharma; meeting participant/lecturer for Astellas, Bayer, Blue Earth Diagnostics, Dendreon Pharmaceuticals, Janssen, Lantheus, Merck, Novartis, Pfizer, Photocure, PROCEPT Biorobotic, Sumitomo Pharma, Tolmar Pharmaceuticals, and UroGen; and scientific study/trial involvement with Astellas, AstraZeneca, Bayer, Dendreon Pharmaceuticals, Immunis.AI, Janssen, Lipella Pharmaceuticals, Merck, Myriad Genetics, Nucleix, Pfizer, and Sumitomo Pharma.

K.A.M. received consulting fees from Boston Scientific and Dendreon Pharmaceuticals.

C.P. reports the following roles: consultant/advisor for AstraZeneca, Bayer, Bristol Myers Squibb, Dendreon, Janssen Oncology, Merck, Pfizer/Astellas, Sun Pharma, Sun Pharma, Tolmar; Speakers’ Bureau for Astellas Pharma, Bayer, and Dendreon; meeting participant/lecturer for Foundation Medicine, Janssen Oncology, Myovant Sciences, Pfizer, and Sun Pharma; and research funding from Advantagene, Astellas Pharma, AstraZeneca, Bayer, Dendreon Pharmaceuticals, Janssen Oncology, Merck, and Pfizer.

B.L. received consulting/research fees from Dendreon Pharmaceuticals.

N.S. reports being employed by Dendreon Pharmaceuticals.

D.J.G. reports the following roles: consultant/advisor for Astellas Pharma, AstraZeneca, Bayer, Dendreon Pharmaceuticals, Exelixis, Janssen, Merck Sharp & Dohme, Michael J. Hennessy Associates, Myovant Sciences, Novartis, Pfizer, and Physicians’ Education Resource; research funding from Astellas Pharma (Inst), AstraZeneca (Inst), Bayer (Inst), Bayer (Inst), Bristol Myers Squibb (Inst), Corvus Pharmaceuticals (Inst), Exelixis (Inst), Janssen Oncology (Inst), Merck (Inst), Novartis (Inst), and Pfizer (Inst).

T.B.D. received consulting fees from Astellas, AstraZeneca, Bayer, Dendreon Pharmaceuticals, Janssen, and Pfizer, and research funds to her institution from AbbVie, Amgen, and AstraZeneca.

## Author Contributions

Neal D. Shore: conceptualization, supervision, and writing–review and editing; Emmanuel S. Antonarakis: writing–review and editing; Jason Hafron: writing–review and editing; Kelvin A. Moses: writing–review and editing; Christopher Pieczonka: writing–review and editing; Benjamin Lowentritt: writing–review and editing; Nadeem Sheikh: conceptualization, supervision, and writing–review and editing; Daniel J. George: writing–review and editing; Tanya Barauskas Dorff: writing–review and editing.

## Funding

This work was sponsored by Dendreon. Dendreon was involved in the design, review, preparation, and approval of and decision to submit the manuscript for publication. The authors not employed by Dendreon were not compensated for their contribution to the manuscript.

## Data Availability

The data that support the findings of this study are available from the corresponding author upon reasonable request.
